# Lesion-specific oral microbiome signatures and predicted carcinogenic pathways in oral squamous cell carcinoma: a paired-site study in Pakistan

**DOI:** 10.1080/20002297.2026.2721025

**Published:** 2026-09-02

**Authors:** Muhammad Irfan Shouq, Hafiz Ghulam Murtaza Saleem, Yuezhu Wang, Muhammad Sohail, Abid Hussain, Huayue Zhang, Huajun Zheng

**Affiliations:** a Department of Medical Lab Technology, Faculty of Rehabilitation & Allied Health Sciences, Riphah International University, Islamabad, Lahore, Pakistan; b Shanghai-MOST Key Laboratory of Health and Disease Genomics, NHC Key Lab of Reproduction Regulation, Shanghai Institute for Biomedical and Pharmaceutical Technologies, Shanghai, China; c National Food Institute, Technical University of Denmark, Lyngby, Denmark

**Keywords:** Oral microbiome, dysbiosis, oral squamous cell carcinoma, 16S rRNA gene, biomarkers, *Prevotella intermedia*, *Segatella oulorum*

## Abstract

**Background:**

Oral squamous cell carcinoma accounts for over 90% of oral neoplasms. Despite therapeutic advances, the lack of reliable, non-invasive biomarkers and delayed diagnosis continues to impede effective clinical management. By combining paired lesion and non-lesion sampling with predictive metagenomics analysis, our study addresses this gap and advances the current understanding of microbiome‒tumor interactions.

**Methods:**

We analyzed 92 buccal swab samples from 39 OSCC patients and 14 healthy controls using 16S rRNA gene (V3–V4) sequencing. Taxonomic profiling was conducted using QIIME2 and SILVA/eHOMD databases, functional pathways were predicted using PICRUSt2, and hub taxa were identified through co-abundance network analysis.

**Results:**

Microbial community structure differed significantly across lesion, non-lesion, and healthy sites (PERMANOVA, *p* = 0.001). Lesions were enriched with *Selenomonas infelix* and *Treponema vincentii*, while healthy controls harbored *Streptococcus oralis* and *Gemella haemolysans*. Co-abundance network analysis revealed lesion-specific hub species, notably *T. vincentii*, strongly correlated with predicted activation of pyrimidine biosynthesis pathways (r = 0.69, q < 1E-6), suggesting predicted metabolic alterations in the tumor microenvironment. Non-lesion sites were also characterized by two hub species, *Prevotella melaninogenica* and *Segatella oulorum*.

**Conclusion:**

Our findings define a lesion-specific microbial signature of OSCC characterized by the depletion of health-associated taxa, enrichment of pro-inflammatory pathobionts, and predicted associations with metabolic pathways implicated in carcinogenesis. These alterations reflect a predicted functionally altered tumor microenvironment.

## Introduction

Oral squamous cell carcinoma (OSCC) ranks as the seventh most common malignancy worldwide and accounts for over 90% of all oral cancers [1]. Global estimates from 2020 reported 377,713 new cases and 177,757 deaths attributable to OSCC [2]. Notably, OSCC represents an increasing oncologic burden in South Asia [2–4]. Despite advancements in therapeutic modalities such as surgery, radiotherapy, and chemotherapy, the five-year survival rate remains disappointingly low at 50–60%, primarily because patients are frequently diagnosed at advanced stages due to a lack of reliable early diagnostic biomarkers [5,6].

OSCC mainly affects middle-aged and older men and can occur in different sites of the oral cavity, including the floor of the mouth, tongue, palate, buccal mucosa, and alveolar ridge [7,8]. The lesions can be asymptomatic/clinically silent at an early stage and then develop into ulcer or nodules with irregular margins [9]. The exact etiology of OSCC is multifactorial, involving a complex interplay between genetic predisposition [10] and environmental risk factors. Established risk factors include alcohol consumption, tobacco use, chewing of betel nuts, nutritional deficiencies, and poor oral hygiene, as well as chronic infections with *Candida*, Epstein–Barr virus, and human papillomavirus [11,12]. A significant proportion of OSCC (15–26%) cases occur in individuals without a history of major risk factors such as tobacco or alcohol. It suggests that less-specified etiological agents are also involved in the pathogenesis and progression of this disease [13].

Emerging evidence now strongly implies that oral microbiome dysbiosis, which is characterized by the disruption of the symbiotic microbial community, is involved in the pathogenesis of OSCC. The oral cavity harbors a diverse microbiota, which, under a state of equilibrium, maintains a symbiotic relationship with the host. Dysbiosis may expose the oral epithelium to microbial virulence factors and carcinogenic metabolites, which can putatively driving aberrant genetic and epigenetic regulations, inhibiting apoptosis, promoting dysplasia and neoplastic transformation [14,15]. This shift in microbial ecology is also observed in oral potentially malignant disorders (OPMDs), including erythroplakia, oral lichen planus, leukoplakia, and verrucous hyperplasia, which carry an increased risk of oncogenic transformation [16,17]. Dysbiosis in both OPMDs and OSCC is often characterized by an enrichment of pathogenic bacteria such as *Fusobacterium nucleatum*, *Prevotella intermedia,* and *Porphyromonas gingivalis* in conjunction with a significant reduction in health-associated commensals [18–20]. The analysis of saliva and buccal swabs offers a cost-effective, non-invasive biofluid medium for microbiome-based diagnostics [21,22]. Furthermore, the development of advanced genomic methods, particularly 16S rRNA gene sequencing and next-generation sequencing (NGS), has enabled the detailed complex oral microbiome characterization and its association with OSCC development [23,24]. While various salivary biomarkers, such as matrix metalloproteinase cytokines, microRNAs, and metabolites, have shown potential for early OSCC detection and prognosis, the potential of microbial signatures has not been fully exploited in certain populations [25].

Despite emergent interest in the role of the oral microbiome in OSCC, most widely available studies have been conducted in Western and East Asian populations, but limited data are available from South Asia. Such a geographical and demographic concentration restricts our understanding of how cultural behaviors, dietary habits, oral hygiene behaviors, and environmental exposures may influence microbiome–tumor interactions in this high-burden region. Notably, few studies have implemented a paired-sampling design, simultaneously analyzing microbiota from tumor lesions, contralateral clinically normal tissue, and healthy controls within the same experimental framework. This lack of spatially resolved comparisons hampers efforts to distinguish cancer-specific microbial alterations from individual variability and compromises the development of valid, novel microbiome-based diagnostic approaches. To comprehensively address these limitations, we performed an extensive microbiome analysis using paired lesion and contralateral non-lesion buccal swabs from patients with OSCC, together with samples from healthy controls. This study aims to delineate lesion-specific microbial communities and their associated metabolic pathways by integrating high-resolution 16S rRNA gene sequencing with predicted functional profiling. In this way, we seek to identify potential associations between microbial composition, the tumor microenvironment and functional capacity, providing a foundation to explore non-invasive and microbiome-informed diagnostic and prognostic candidates that may be employed across global populations.

## Materials and methods

### Sample collection

A total of 92 buccal swab samples were collected for this study, comprising 78 samples from patients with histopathologically confirmed OSCC and 14 samples from healthy control subjects (HC). Paired swabs were obtained, one from the tumor lesion site (lesion-site) and one from the contralateral, clinically healthy, non-lesion buccal mucosa (contralateral non-lesion). All OSCC cases were recruited from the Department of Oral and Maxillofacial–Head and Neck Oncology at Mayo Hospital and INMOL Cancer Hospital, Lahore, Pakistan.

Samples were collected using sterile nylon-flocked swabs (CY-98000T, Hua Chenyang Technology Co., Ltd., Shenzhen, China) according to a standardized clinical protocol. For lesion sites, the swab was gently rubbed and rotated 10–15 times over the surface of the tumor. The same process was done to the contralateral, anatomically matched non-lesion site of OSCC to ensure consistency in sampling.

Before sample collection, all participants were advised to avoid drinking, eating, smoking, or performing oral hygiene for at least 2 h. Moreover, OSCC patients with a history of antibiotic use within 1 week before sampling were excluded to avoid short-term microbiome disruption. Each swab was placed immediately after collection in a sterile, nuclease-free tube comprising DNA preservation buffer to ensure genomic integrity. The samples were then kept at −20°C until further processing for DNA extraction.

### Genomic DNA extraction, PCR amplification and gene sequencing

Using the Hi-swab DNA Extraction kit (TIANGEN, China), the total genomic DNA was extracted from the buccal swab samples, according to the manufacturer’s protocol. The hypervariable V3–V4 region of the bacterial 16S rRNA gene was amplified by polymerase chain reaction (PCR) using universal primers 338F (5´-ACTCCTACGGGAGGCAGCAG-3´) and 806R (5´-GGACTACHVGGGTWTCTAAT-3´) [26]. PCR amplification was performed using TransStart FastPfu DNA Polymerase (TransGen, China). The thermocycling conditions were optimized as follows: an initial denaturation at 95°C for 2 min; 20 cycles of denaturation at 95°C for 45 s, annealing at 55°C for 30 s, and extension at 72°C for 30 s; followed by a final extension at 72°C for 5  min [27].

During DNA extraction, a blank swab control (sterile nylon-flocked swab processed throughout the entire extraction workflow without patient contact) was included in each batch; no DNA was detectable in these blank controls by agarose gel electrophoresis. Additionally, a no-template PCR negative control (nuclease-free water replacing template DNA) was included in each PCR amplification batch; no amplification product was observed in these negative controls. However, these controls were not subjected to downstream sequencing, which precludes statistical decontamination of the sequenced samples.

The amplicon quality was verified by electrophoresis on a 2% agarose gel, and the target bands were purified using the AxyPrep DNA Gel Extraction Kit (AXYGEN, USA). Equimolar concentrations of purified PCR products were pooled to construct a single sequencing library, which was sequenced on a Salus Pro platform (Salus, China) using a paired-end 2× 300  bp strategy.

### Bioinformatics and statistical analysis

Raw sequencing data in FASTQ format were processed using QIIME2 (version 2024.5). Quality control, denoising, merging of paired-end reads, and chimera removal were performed using the DADA2 plugin within QIIME2 to obtain high-quality amplicon sequence variants (ASVs) [28]. The forward and reverse reads were truncated at 280 bp and 220 bp, respectively, based on quality score profiles; all other parameters were kept at default. Alpha diversity, representing within-sample diversity, was assessed using metrics of richness (ACE index), evenness (Shannon’s evenness), and overall diversity (Shannon index), based on normalized ASV tables (using 78,173 as the minimum reads). Alpha diversity comparisons across the three groups (HC, lesion-site, and non-lesion) were assessed using the Kruskal‒Wallis H test. When the global test was significant, pairwise comparisons were performed using the Mann‒Whitney U test with Benjamini‒Hochberg (BH) correction for multiple comparisons. For the paired comparison between lesion-site and contralateral non-lesion samples from the same subject (*n* = 39 pairs), the Wilcoxon signed-rank test was employed to account for within-subject dependence. Statistical significance was set at the BH-adjusted *p*-value (q) <  0.05 for Mann–Whitney tests and *p* <  0.05 for the Wilcoxon signed-rank tests.

Permutational multivariate analysis of variance (PERMANOVA) based on Bray‒Curtis dissimilarity was used to test beta diversity, comparing the differences in microbial community structure between groups, implemented in PAST software (version 5.2) [29]. To account for the paired sampling design, subject ID was included as a stratification factor (blocking variable) in the permutation procedure for comparisons involving lesion-site and contralateral non-lesion paired samples. Statistical significance was assessed using 9,999 permutations in all tests. Effect sizes were reported as R^2^ values, representing the proportion of variance in community composition explained by group membership.

Taxonomic assignment of ASVs was performed using the RDP Classifier (version 2.14) [30] with a confidence threshold of 80%. For refined species-level identification, ASVs were further queried using BLASTN against the SILVA database (version 138.2) [30] and the expanded Human Oral Microbiome Database (eHOMD, version 16.01) [31], retaining hits with sequence identity and alignment coverage scores greater than 97%. Functional profiling of the microbial communities was inferred from 16S rRNA data using the PICRUSt2 pipeline [32], after normalizing for gene copy number. Pathway annotations were obtained from the MetaCyc database. Differential abundance analysis of taxa between groups was performed using MaAsLin3 (microbiome multivariable associations with linear models) [33]. The analysis was configured as follows: (i) fixed effect: sample group (lesion-site, contralateral non-lesion, healthy control); (ii) random effect: subject ID; (iii) prevalence filter: taxa present in > = 10% of samples; (iv) abundance filter: minimum relative abundance > 0.005%; (v) normalization: total-sum scaling followed by asin-square-root transformation; (vi) zero-handling: MaAsLin3's default two-part model; (vii) significance threshold: adjusted *p*-value (q) < 0.1. Subject was included as a random effect to account for within-subject correlation between lesion-site and contralateral non-lesion paired samples, and taxa with an adjusted *p*-value < 0.1 (default significance threshold) and a minimum relative abundance > 0.0005 were considered statistically significant.

To assess the robustness of differential abundance findings across taxonomic resolutions, a confirmatory sensitivity analysis was performed at the genus level using the Mann‒Whitney U test with Benjamini‒Hochberg (BH) false discovery rate (FDR) correction. Relative abundances were compared between lesion-site and healthy-control samples, between lesion-site and contralateral non-lesion samples, and between contralateral non-lesion and healthy-control samples, respectively. Statistical significance was set at the BH-adjusted *p*-value (q) < 0.05.

Co-abundance networks were constructed by calculating robust correlations between microbial species using FastSpar [34]. Only correlations with a Spearman's coefficient > |0.5| and a Benjamini–Hochberg (BH)-adjusted *p*-value < 0.05 were retained for network construction. Hub species were identified using the cytoHubba plugin [35] of Cytoscape (version 3.10.3) [36] based on a consensus of six centrality algorithms: maximum clique centrality (MCC), maximum neighborhood component (MNC), degree, closeness, radiality, and edge percolated component (EPC). Finally, Spearman correlation analysis was used to assess associations between the abundance of hub species and predicted MetaCyc pathway abundances. Associations with a correlation coefficient > |0.5| and BH-adjusted *p*-value < 0.05 were considered significant.

Five machine-learning algorithms, support vector machine (SVM), decision tree (DT), random forest (RF), k-nearest neighbor (KNN) and logistic regression (LR), were employed to explore candidate microbial markers for distinguishing OSCC from healthy controls (HC) in an exploratory analysis. All the models were implemented in scikit-learn. ASV abundances were CLR-transformed (pseudocount = 0.5) within each cross-validation (CV) fold to prevent data leakage. Feature selection (SelectKBest, ANOVA F-score) was nested within each CV training fold, with k ∈ [4, 20] optimized via inner validation. Model performance was evaluated using repeated stratified 5-fold CV (10 repeats, 50 evaluations per classifier), with class imbalance addressed by class_weight = 'balanced' (SVM, LR, RF, and DT) or distance weighting (KNN). All the metrics (AUC, accuracy, sensitivity, and specificity) are reported as mean ± SD. SHAP (SHapley Additive exPlanations) analysis was performed on the final RF model to interpret feature contributions.

## Results

### Participant characteristics

This study enrolled 39 patients with a confirmed diagnosis of OSCC. The median age of the cohort was 49 years, predominantly male (78.0%). The socioeconomic and demographic assessment showed that more than half of the study population was illiterate (58.5%), and the large majority (87.8%) belonged to a lower socioeconomic group. Almost half (48.8%) were in unskilled labor occupations.

In the context of behavioral risk factors, betel quid consumption was the most commonly observed (87.8%), followed by tobacco use (36.6%). Clinically, the most frequent presenting symptoms were identified to be the presence of an oral ulcer (85.4%) and difficulty in swallowing (78.0%). The comprehensive demographic and clinical characteristics of the cohort are summarized (Table 1).

**Table 1.** Sociodemographic and clinical characteristics of patients with oral squamous cell carcinoma (OSCC) (*n* = 39).

Characteristic	Category	*n* (%)
Age (years)		Median [IQR] or Frequency: 49 [37,38]
Gender	Male	30 (77.0)
	Female	9 (23.0)
Education	Illiterate	22 (56.4)
	Primary (1st-5th)	10 (24.4)
	Middle (6th-8th)	4 (9.8)
	Secondary (9th-12th)	3 (7.3)
	Graduate+	0 (0.0)
Occupation	Laborer	18 (46.1)
	Housewife	8 (19.5)
	Driver	4 (9.8)
	Workers with technical skills	5 (12.2)
	Other	4 (9.8)
Residence	Urban	22 (56.4)
	Rural	17 (41.5)
Socioeconomic Status	Poor	34 (87.1)
	Rich	5 (12.2)
Tobacco Use	Yes	15 (36.6)
	No	24 (61.5)
Alcohol Use	Yes	7 (17.1)
	No	32 (82.0)
Betel Quid Use	Yes	34 (87.1)
	No	5 (12.2)
Family History of Cancer	Yes	8 (19.5)
	No	31 (79.4)
Previous Dental Visit	Yes	22 (53.7)
	No	17 (43.5)
Oral Ulcer	Yes	33 (84.6)
	No	6 (14.6)
Difficulty in Swallowing	Yes	30 (76.9)
	No	9 (22.0)

### Cancer grading and staging distribution

All 39 cases of OSCC were graded and staged based on the 8th Edition TNM classification system of the American Joint Committee on Cancer (AJCC). The distribution of tumor stages and histological grades in the patient cohort is presented (Table 2).

**Table 2.** Cancer stages and grade-wise distribution of OSCC patients (*n* = 39 patients).

Stage	Classification	Patients *n* (%)
I	Early localized	2 (4.8)
II	Local advancement	6 (15.3)
III	Regional spread	10 (24.3)
IV	Advanced metastasis	21 (53.8)
**Grade**	**Differentiation**	**Patients**
G1	Well-differentiated	24 (61.5)
G2	Moderately differentiated	13 (33.3)
G3	Poorly differentiated	2 (4.8)

### Characteristics of microbiota composition and abundance

A total of 18.5 million pairs of reads were produced for 92 oral samples. After quality control and denoising, 16,766,231 high-quality sequences were obtained to characterize the oral microbiota across different sites. A total of 5,282 amplicon sequence variants (ASVs) were annotated after bioinformatic processing and normalization, spanning 18 phyla, 34 classes, 68 orders, 147 families, and 344 genera (Supplementary Table 1). The rarefaction curves demonstrated that a saturation number of ASVs had been approached in each sample (Supplementary Figure 1).

Phylum-level analysis demonstrated that the oral microbiota was markedly composed of Bacillota (average relative abundance: 33.57%), Bacteroidota (24.00%), Pseudomonadota (22.95%), Fusobacteriota (10.02%), and Actinomycetota (4.33%) (Table 3 for phyla with > 0.10% abundance in at least one site).

**Table 3.** The dominant taxa in the oral cavity.

Level	Feature	Total	Lesion-site	Non-lesion	HC
**phylum**	Bacillota	33.57%	17.33%	42.93%	52.73%
**phylum**	Bacteroidota	24.00%	37.11%	16.54%	8.28%
**phylum**	Pseudomonadota	22.95%	20.29%	25.48%	23.32%
**phylum**	Fusobacteriota	10.20%	15.58%	6.35%	5.94%
**phylum**	Actinomycetota	4.33%	1.27%	6.05%	8.10%
**phylum**	Spirochaetota	2.03%	3.99%	0.67%	0.36%
**phylum**	Campylobacterota	1.44%	2.55%	0.72%	0.38%
**phylum**	Candidatus Saccharibacteria	0.69%	0.68%	0.72%	0.65%
**phylum**	Mycoplasmatota	0.39%	0.81%	0.11%	0.03%
**phylum**	unclassified_Bacteria	0.19%	0.17%	0.25%	0.09%
**phylum**	SR1	0.12%	0.17%	0.08%	0.09%
**genus**	*Streptococcus*	20.68%	5.44%	28.33%	41.80%
**genus**	*Neisseria*	7.74%	6.15%	10.31%	4.98%
**genus**	*Haemophilus*	7.41%	4.60%	9.12%	10.48%
**genus**	*Gemella*	3.57%	1.19%	4.89%	6.51%
**genus**	*Prevotella*	7.42%	12.57%	4.61%	0.91%
**genus**	*Fusobacterium*	7.48%	11.78%	4.37%	4.14%
**genus**	*Rothia*	2.28%	0.30%	3.37%	4.77%
**genus**	*Porphyromonas*	3.96%	5.21%	3.13%	2.81%
**genus**	*Granulicatella*	1.87%	0.69%	3.12%	1.65%
**genus**	*Capnocytophaga*	4.70%	7.88%	2.91%	0.83%
**genus**	*Veillonella*	2.74%	3.40%	2.54%	1.44%
**genus**	*Leptotrichia*	2.12%	2.74%	1.75%	1.41%
**genus**	*Aggregatibacter*	2.53%	3.75%	1.20%	2.85%
**genus**	*Actinomyces*	0.88%	0.16%	1.17%	2.09%
**genus**	*Segatella*	1.29%	2.19%	0.79%	0.21%
**genus**	*Campylobacter*	1.31%	2.23%	0.72%	0.38%
**genus**	*Treponema*	2.00%	3.93%	0.67%	0.35%
**species**	*Streptococcus oralis*	15.37%	2.62%	21.77%	33.05%
**species**	*Prevotella intermedia*	3.90%	7.37%	1.74%	0.23%
**species**	*Haemophilus haemolyticus*	3.72%	1.96%	4.86%	5.48%
**species**	*Gemella haemolysans*	3.23%	0.77%	4.58%	6.33%
**species**	*Haemophilus parainfluenzae*	2.86%	2.34%	3.35%	2.97%
**species**	*Neisseria mucosa*	2.84%	1.76%	4.34%	1.67%
**species**	*Fusobacterium polymorphum*	2.34%	4.02%	1.26%	0.67%
**species**	*Capnocytophaga leadbetteri*	2.14%	3.83%	1.14%	0.24%
**species**	*Prevotella melaninogenica*	2.11%	2.64%	2.23%	0.32%
**species**	*Neisseria perflava*	2.11%	1.52%	2.79%	1.86%
**species**	*Fusobacterium periodonticum*	1.88%	2.97%	0.95%	1.46%
**species**	*Aggregatibacter segnis*	1.88%	3.13%	0.68%	1.76%
**species**	*Streptococcus gordonii*	1.77%	1.23%	2.69%	0.76%
**species**	*Fusobacterium hwasookii*	1.55%	2.56%	0.92%	0.47%
**species**	*Veillonella parvula*	1.45%	1.98%	1.26%	0.48%
**species**	*Streptococcus mitis*	1.44%	0.09%	1.06%	6.25%
**species**	*Rothia dentocariosa*	1.39%	0.03%	1.86%	3.86%
**species**	*Porphyromonas endodontalis*	1.10%	1.90%	0.56%	0.40%
**species**	*Porphyromonas pasteri*	1.06%	0.79%	1.36%	0.99%
**species**	*Lautropia mirabilis*	1.01%	0.12%	1.74%	1.45%
**species**	*Porphyromonas gingivalis*	0.96%	1.91%	0.15%	0.53%
**species**	*Granulicatella adiacens*	0.95%	0.51%	1.63%	0.28%
**species**	*Granulicatella elegans*	0.92%	0.18%	1.49%	1.37%
**species**	*Capnocytophaga granulosa*	0.91%	1.40%	0.68%	0.16%
**species**	*Neisseria cinerea*	0.89%	0.51%	1.45%	0.38%
**species**	*Capnocytophaga sputigena*	0.76%	1.26%	0.48%	0.15%
**species**	*Neisseria elongata*	0.70%	1.26%	0.33%	0.18%
**species**	*Fusobacterium watanabei*	0.70%	1.18%	0.37%	0.29%
**species**	Aggregatibacter aphrophilus	0.65%	0.61%	0.52%	1.09%
**species**	Treponema vincentii	0.61%	1.27%	0.17%	0.03%
**species**	Prevotella nigrescens	0.61%	1.28%	0.13%	0.07%
**species**	Alloprevotella tannerae	0.56%	1.05%	0.24%	0.09%
**species**	Treponema denticola	0.55%	1.22%	0.07%	0.05%
**species**	Haemophilus parahaemolyticus	0.54%	0.15%	0.69%	1.21%
**species**	Segatella oris	0.54%	1.01%	0.21%	0.13%
**species**	Campylobacter gracilis	0.54%	1.00%	0.25%	0.05%
**species**	Enterobacter hormaechei	0.49%	1.15%	0.01%	0.01%
**species**	Pseudomonas aeruginosa	0.48%	1.06%	0.06%	0.01%

At smaller scales, there were taxonomic changes. At the genus level, 17 dominant genera (each with > 2.00% relative abundance in at least one site) cumulatively constituted for 79.98% of the total microbiota. Each of these 17 core genera was consistently present in all the healthy control samples. The putative species-level analysis highlighted that the predominant species in the healthy controls (HC) and non-lesion sites were *Streptococcus oralis* (33.05 and 21.77%, respectively) and *Gemella haemolysans* (6.33 and 4.86%, respectively). In comparison, the lesion sites were enriched in *Prevotella intermedia* (7.37%).

**Figure 1.**
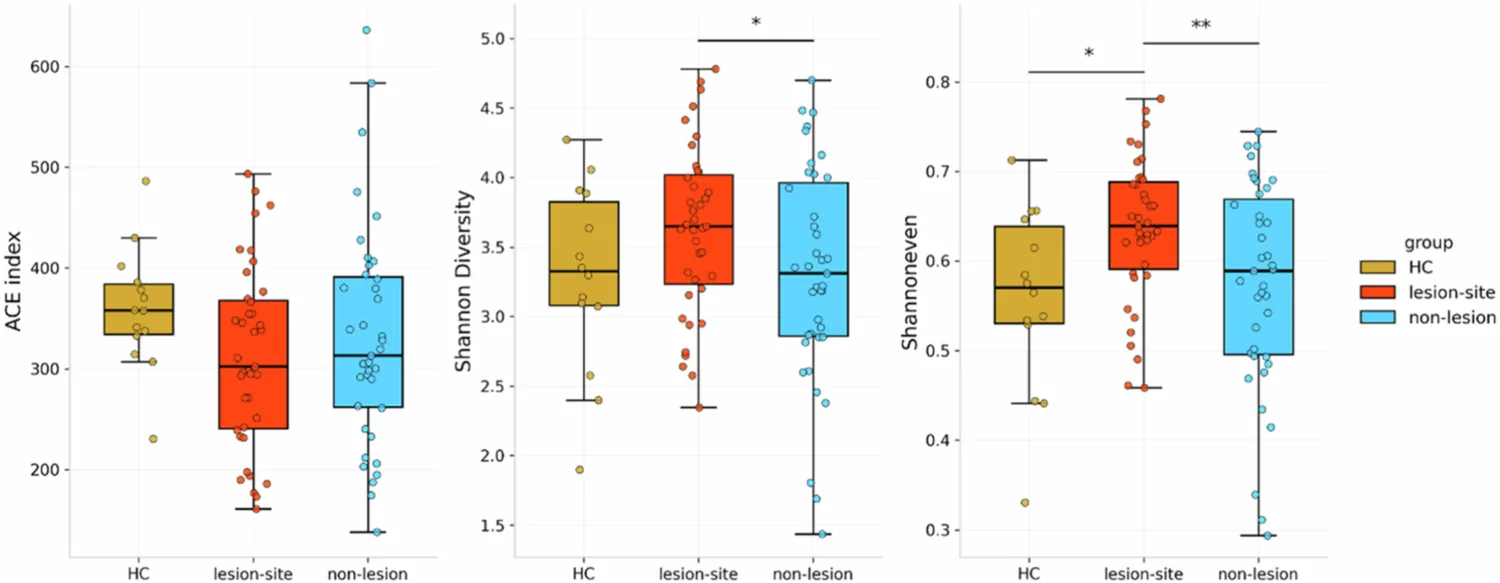
Alpha diversity indices (ACE richness, Shannon diversity and Shannon evenness) among HC, lesion-site and non-lesion groups. HC, oral microbiota of healthy controls; lesion-site, microbiota of tumor lesion sites from OSCC patients; non-lesion, microbiota of contralateral, non-lesion buccal mucosa from OSCC patients. The “*” indicates *p* < 0.05, and “**” indicates *p* < 0.01.

The alpha diversity was determined using the ACE (richness), Shannon's evenness (evenness), and Shannon (diversity) indices (Figure 1). The microbial richness (ACE index) tended to be greater at the HC sites compared to both lesion and non-lesion sites, although this difference did not reach statistical significance (Kruskal‒Wallis H = 3.69, *p* = 0.158). Overall diversity (Shannon index) exhibited a significant increase at lesion sites relative to non-lesion sites in the paired comparison (Wilcoxon signed-rank V = 212.0, *p* = 0.012). Microbial evenness (Shannon evenness index) was significantly higher at lesion sites compared to both HC (Mann‒Whitney U = 150.0, BH-adjusted *p* = 0.034) and non-lesion sites (paired Wilcoxon V = 182.0, *p* = 0.003).

**Figure 2.**
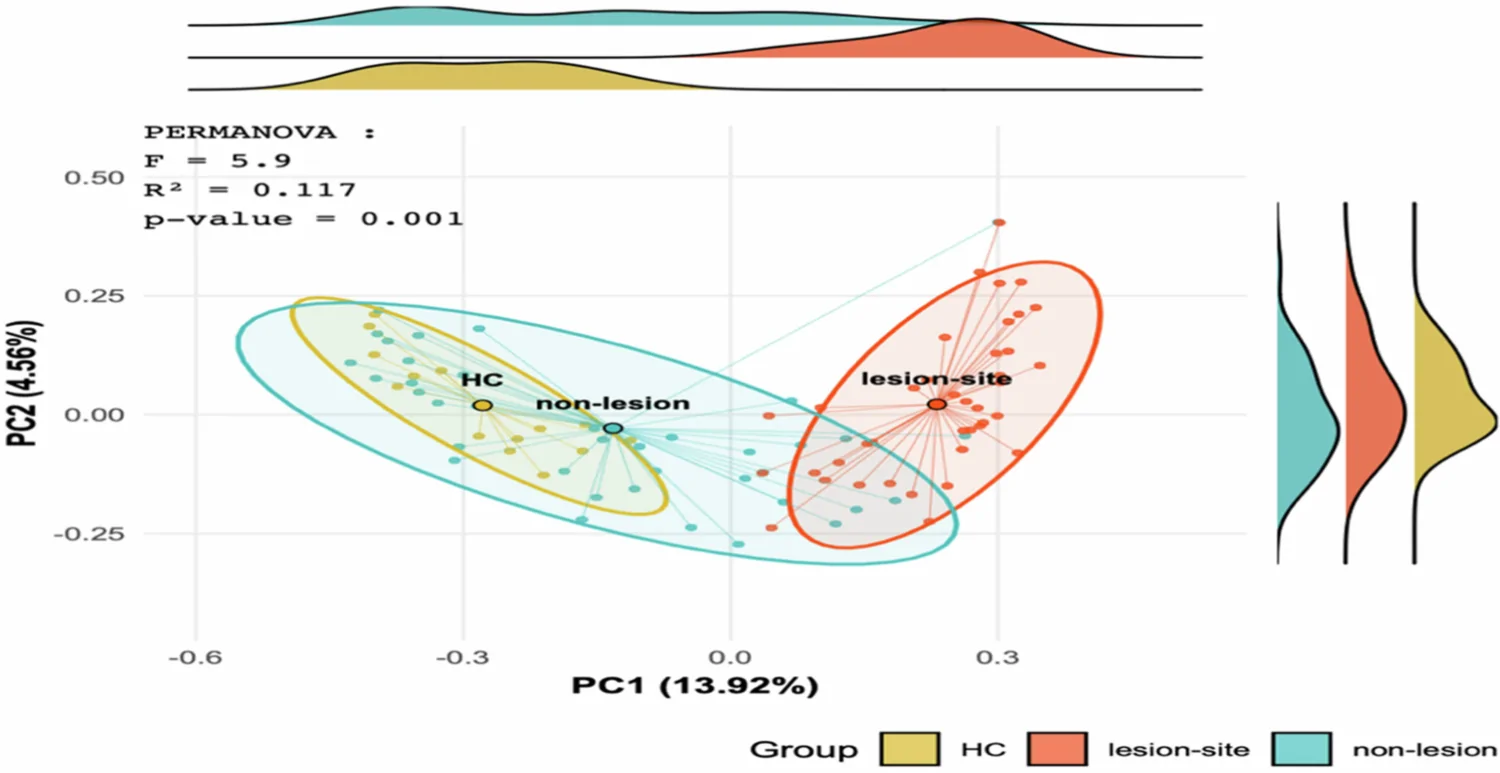
Community structure shown by principal coordinate analysis (PCoA) of oral microbiota based on Bray–Curtis dissimilarity. Statistical significance was assessed by PERMANOVA with subject-stratified permutation (9,999 permutations). Global test: F = 5.90, R^2^ = 0.117, *p* = 0.001. Pairwise comparisons – lesion-site vs non-lesion: F = 7.03, R^2^ = 0.085, *p* < 0.001; lesion-site vs HC: F = 7.22, R^2^ = 0.124, *p* < 0.001; non-lesion vs HC: F = 1.99, R^2^ = 0.038, *p* = 0.007.

Bray–Curtis distance-based PERMANOVA revealed that the overall composition of the microbial community differed significantly between the three sample groups (*p* = 0.001). The principal coordinate analysis (PCoA) provided visual evidence to support this observation, as the analysis revealed a clear separation of microbial profiles, especially for the lesion sites (Figure 2).

Despite some overlap in the microbial community of HC and non-lesion sites in the PCoA plot, their compositions remained statistically different (PERMANOVA, *p* = 0.007).

Collectively, these findings point to the fact that the microbial community structure at lesion sites differs fundamentally from that of its contralateral non-lesion tissue and healthy control mucosa.

### Microbiota composition differences of patients with OSCC

We performed a comparative analysis of taxonomic abundances at the phylum, genus, and putative species levels across the three sample groups to elucidate the microbial communities associated with OSCC progression. There were considerable changes across all the taxonomic levels. A significant statistical difference in abundance across the lesion, non-lesion, and healthy control (HC) sites was found in seven phyla, 25 genera, and 55 species (Supplementary Table 2).

**Figure 3.**
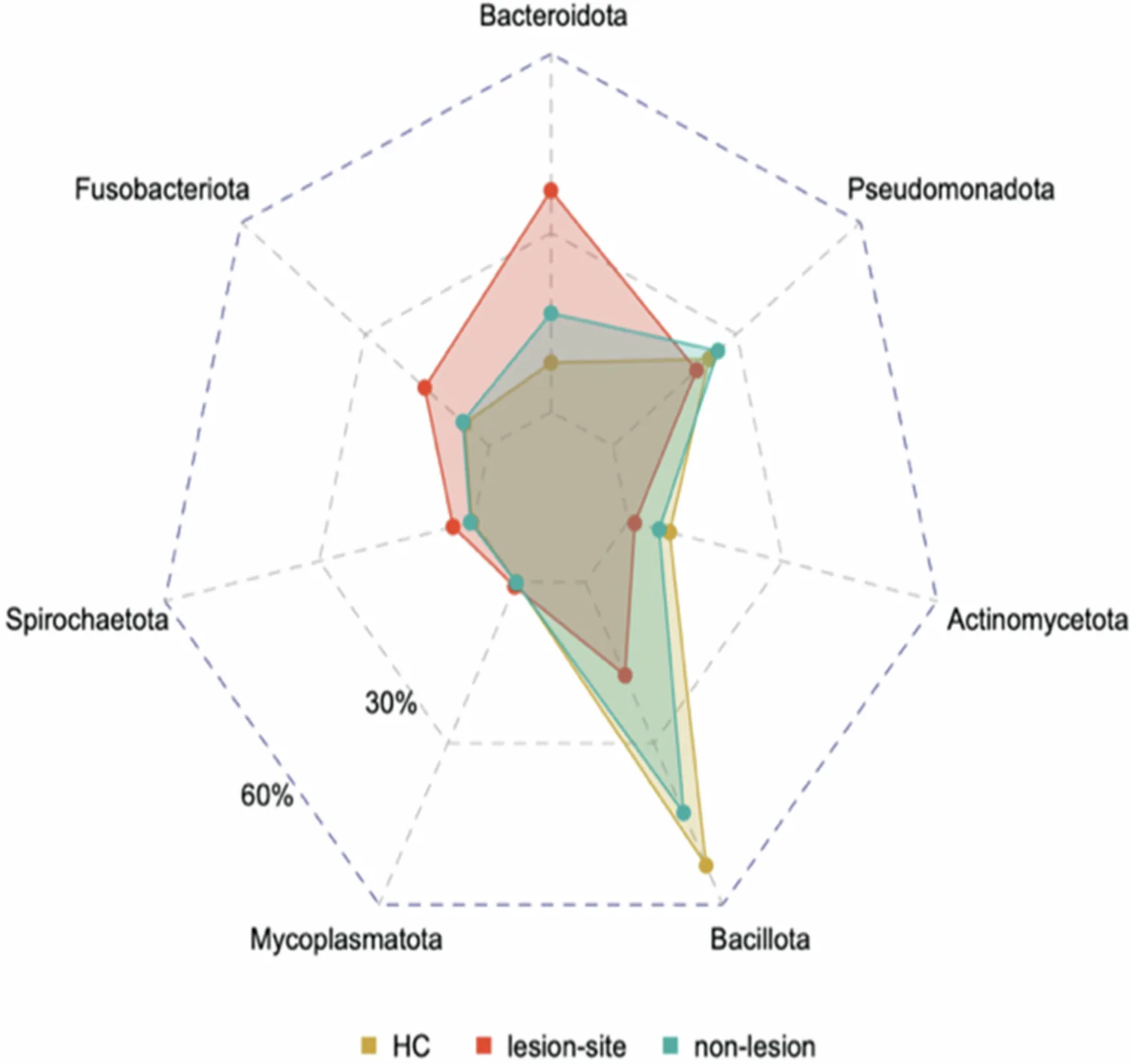
Significantly changed phyla of oral microbiota across three sites (HC, lesion-site, and non-lesion). HC, oral microbiota of healthy controls; lesion-site, microbiota of tumor lesion site from OSCC patients; non-lesion, microbiota of contralateral, non-lesion buccal mucosa from OSCC patients.

At the phylum level, lesion sites were significantly enriched with the Bacteroidota (37.11%) as compared to non-lesion sites (16.54%) and healthy controls (8.28%). In contrast, Bacillota was significantly enriched in healthy controls (54.73%) compared with both lesion (17.33%) and non-lesion (42.93%) sites (Figure 3).

**Figure 4.**
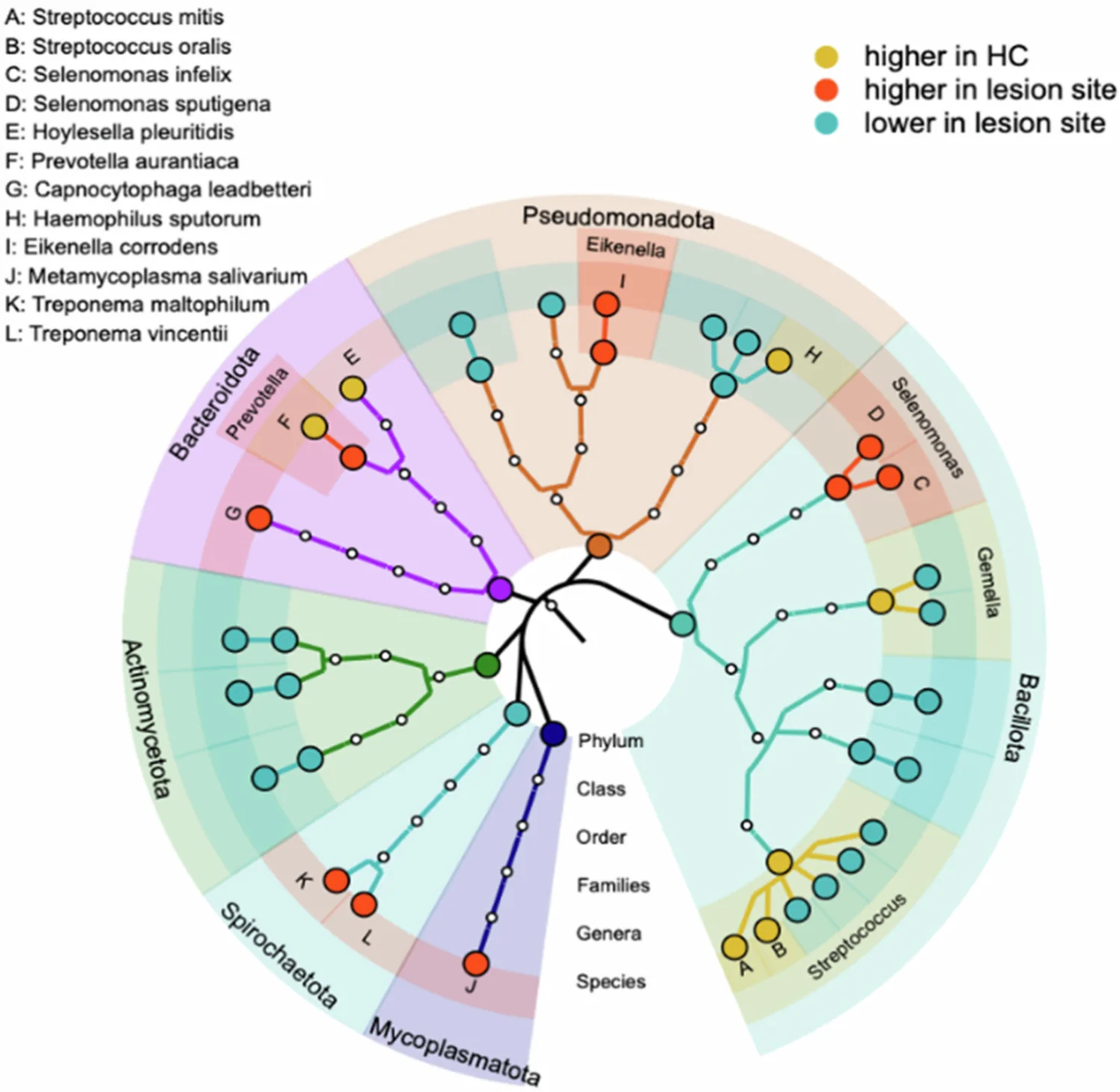
Significantly changed genus and putative species of oral microbiota across three sites. Differential abundance of oral microbiota across sites. Features are categorized as significantly “higher in HC” (vs. both OSCC sites), “higher in lesion site” (vs. non-lesion and HC), or “lower in lesion site” (vs. non-lesion and HC). The label showed putative species descriptions of “higher in HC” and “higher in lesion site”. HC, oral microbiota of healthy controls; lesion-site, microbiota of tumor lesion site from OSCC patients; non-lesion, microbiota of contralateral, non-lesion buccal mucosa from OSCC patients.

Genus-level analysis showed a consistent pattern of dysbiosis. Lesion sites were significantly enriched in the genera *Prevotella, Selenomonas*, and *Eikenella* as compared to both non-lesion and healthy control sites. Conversely, *Gemella* and *Streptococcus* were markedly decreased in lesion and non-lesion sites, demonstrating their highest abundance in healthy controls (Figure 4). Genus-level sensitivity analyses confirmed the robustness of these findings: the same directional trends were observed at q < 0.05 (Mann‒Whitney U test with Benjamini‒Hochberg correction), with 18 genera significantly different between lesion and healthy-control sites and 20 between lesion and non-lesion sites (Supplementary Table 3). Notably, no genus was significantly different between contralateral non-lesion and healthy-control sites at q < 0.05, supporting the interpretation that microbial dysbiosis is primarily driven by the lesion microenvironment rather than by patient-level confounders.

Key taxonomic drivers of this dysbiotic state were found with analysis at the putative species level. Seven species were significantly enriched in lesion sites, including *Selenomonas infelix*, *Treponema vincentii*, *Capnocytophaga leadbetteri*, *Treponema maltophilum*, *Selenomonas sputigena*, *Eikenella corrodens*, and *Metamycoplasma salivarium*. In contrast, five species, *Streptococcus oralis*, *Prevotella aurantiaca, Streptococcus mitis*, *Haemophilus sputorum* and *Hoylesella pleuritidis,* were significantly depleted in OSCC patients, showing their highest abundance in healthy controls. An additional 15 species, such as *Gemella haemolysans*, were much lower in lesion sites compared with both non-lesion and HC sites. Taken together, these findings indicate a profound and structured shift in the oral microbiota, which is closely linked to OSCC, characterized by the enrichment of specific pathobionts and the depletion of health-associated commensals (Figure 4).

### Microbiota biomarkers distinguishing HC and patients with OSCC

**Figure 5.**
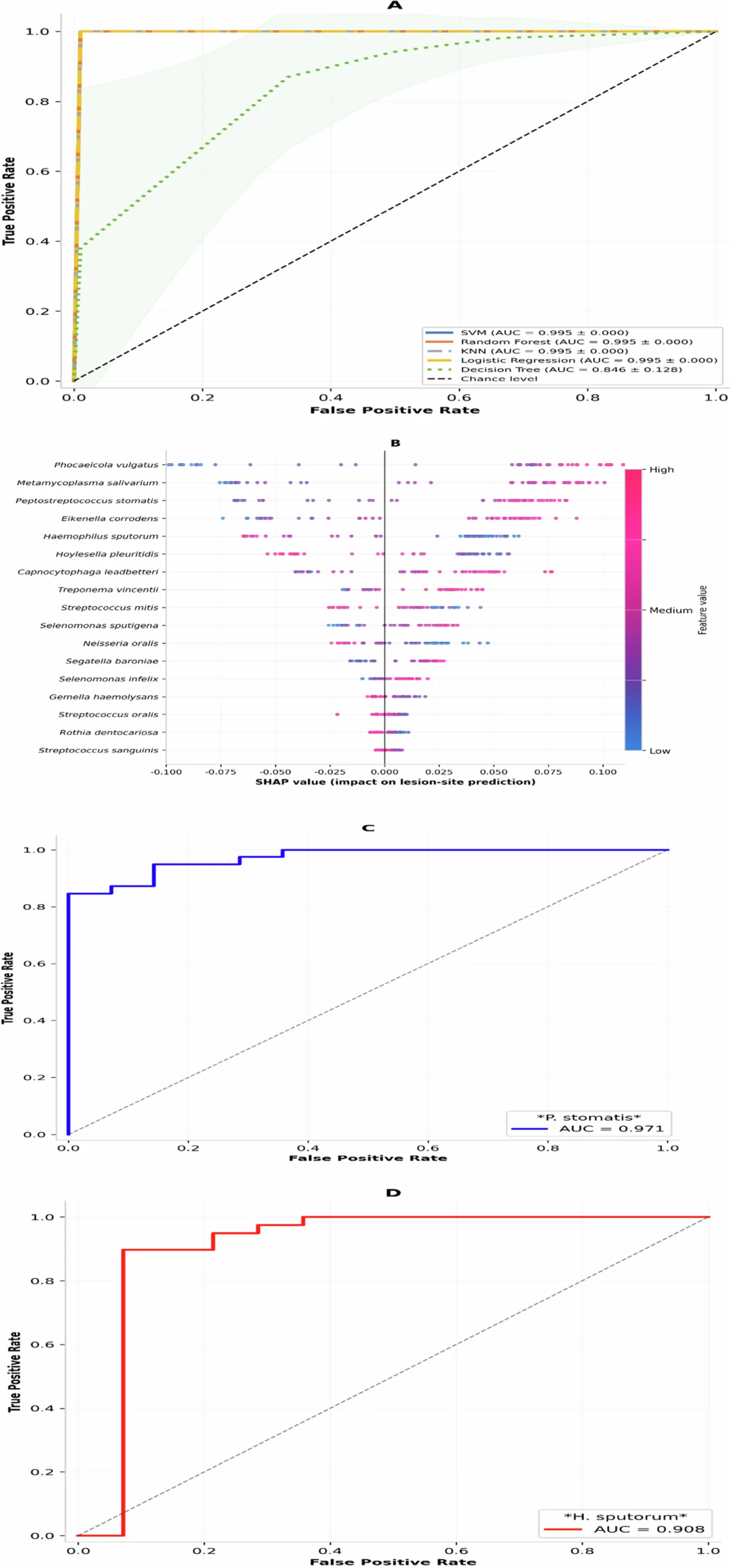
Diagnostic performance and SHAP-based identification of key microbial biomarkers distinguishing OSCC lesion-site and healthy control samples. (A) Receiver operating characteristic (ROC) curves for five machine-learning classifiers under leakage-free nested repeated stratified 5-fold cross-validation. Solid lines represent the mean true positive rate across 50 evaluations; shaded regions indicate ± 1 SD. The legend values indicate the AUC of the mean ROC curve (individual fold AUCs: SVM/RF/KNN/LR = 1.000 ± 0.000 each; DT = 0.848 ± 0.128). (B) SHAP summary plot showing the most influential microbial species ranked by the mean absolute SHAP value. Colors represent CLR-transformed abundance (blue = low, pink/red = high). Positive SHAP values indicate contribution toward lesion-site classification; negative values indicate contribution toward healthy control classification. (C) ROC curve demonstrating the discriminative performance of *Peptostreptococcus stomatis* between lesion-site and healthy control samples (AUC = 0.971). (D) ROC curve demonstrating the discriminative performance of *Haemophilus sputorum* between healthy control and lesion-site samples (AUC = 0.908). RF, random forest; SVM, support vector machine; DT, decision tree; KNN, k-nearest neighbor; LR, logistic regression. HC, oral microbiota of healthy controls; lesion-site, microbiota of tumor lesion-site from OSCC patients.

To verify species enriched in HC (27 species) or in lesion-site samples (11 species), five machine-learning methods with nested, leakage-free cross-validation were used to explore their discriminative potential in an exploratory candidate biomarker analysis (Figure 5A). Among all the models, the RF classifier achieved the highest performance across four evaluation metrics – AUC, accuracy, sensitivity, and specificity – followed by SVM and LR. A permutation test (1,000 permutations) confirmed the statistical significance of RF performance (empirical *p* < 0.001). Based on RF, we selected nine HC-enriched species and eight lesion-site-enriched species with scores > 0.8 (across all four indicators) for SHAP interpretation.

SHAP analysis highlighted the most influential biomarkers. For the lesion-site samples, the top contributors were *Phocaeicola vulgatus*, *Metamycoplasma salivarium*, *Peptostreptococcus stomatis*, *Eikenella corrodens*, *Capnocytophaga leadbetteri* and *Treponema vincentii*, among others (Figure 5B). Among them, *P. stomatis* demonstrated the highest discriminative ability between HC and lesion sites, with an area under the curve (AUC) of 0.971 (Figure 5C). The most important species associated with healthy control samples included *Haemophilus sputorum*, *Streptococcus mitis*, *Neisseria oralis*, and *Streptococcus oralis*, among others (Figure 5B), and *H. sputorum* achieved the highest AUC of 0.908 for distinguishing HC from lesion sites (Figure 5D).

### Microbiota species correlations and functional profiling

**Figure 6.**
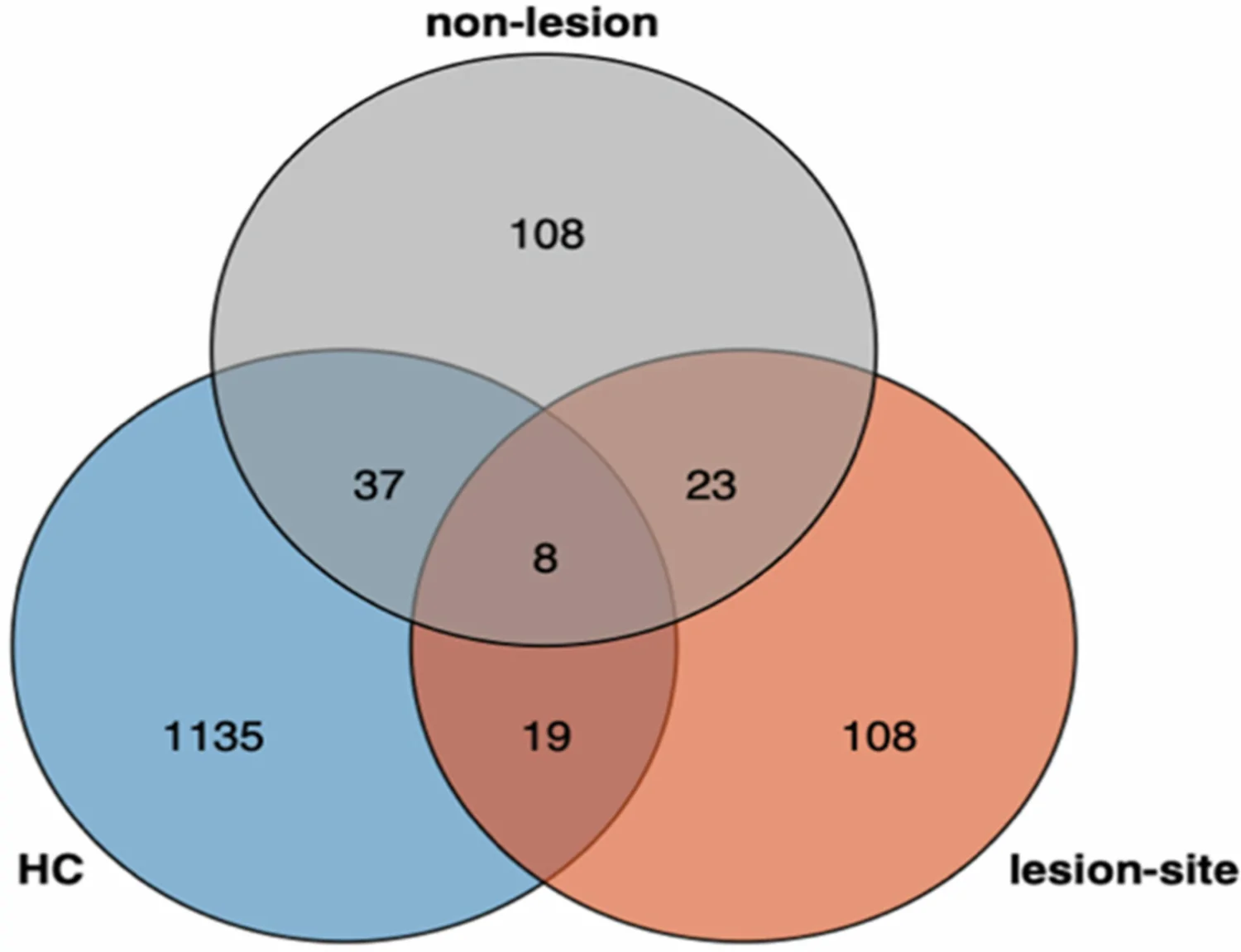
Venn diagram illustrating the number of significant positive correlations shared among HC, lesion-site and non-lesion sites. HC, oral microbiota of healthy controls; lesion-site, microbiota of tumor lesion site from OSCC patients; non-lesion, microbiota of contralateral, non-lesion buccal mucosa from OSCC patients.

We constructed co-abundance networks and identified hub species separately for the healthy control (HC), non-lesion and lesion sites (FDR-adjusted q < 0.05) to delineate the complex microbial interactions within the oral microbial ecology. The complexity of the network was highest in HC, where 208 species formed 1,199 significant correlations (796 positive, 403 negative). Conversely, the networks for lesion and contralateral non-lesion sites were less complex, consisting of 115 species with 158 correlations and 114 species with 176 correlations, respectively. A comparative analysis showed that eight conserved positive correlations were shared among all three sites (Figure 6), including a strong positive relationship between *Gemella haemolysans and Streptococcus oralis,* which are the two most abundant commensals in healthy controls.

Hub species analysis, which identifies highly connected keystone taxa, revealed distinct leaders for each community. In healthy controls, the hub species were *Corynebacterium durum*, *Streptococcus sanguinis*, *Peptidiphaga gingivicola* and *Rothia aeria*. Conversely, the lesion site network was dominated by Treponemes: *Treponema medium*, *Treponema socranskii,* and *Treponema vincentii*, the latter of which was also significantly enriched in lesion sites. The non-lesion site network was characterized by two hub species: *Prevotella melaninogenica* and *Segatella oulorum*.

**Figure 7.**
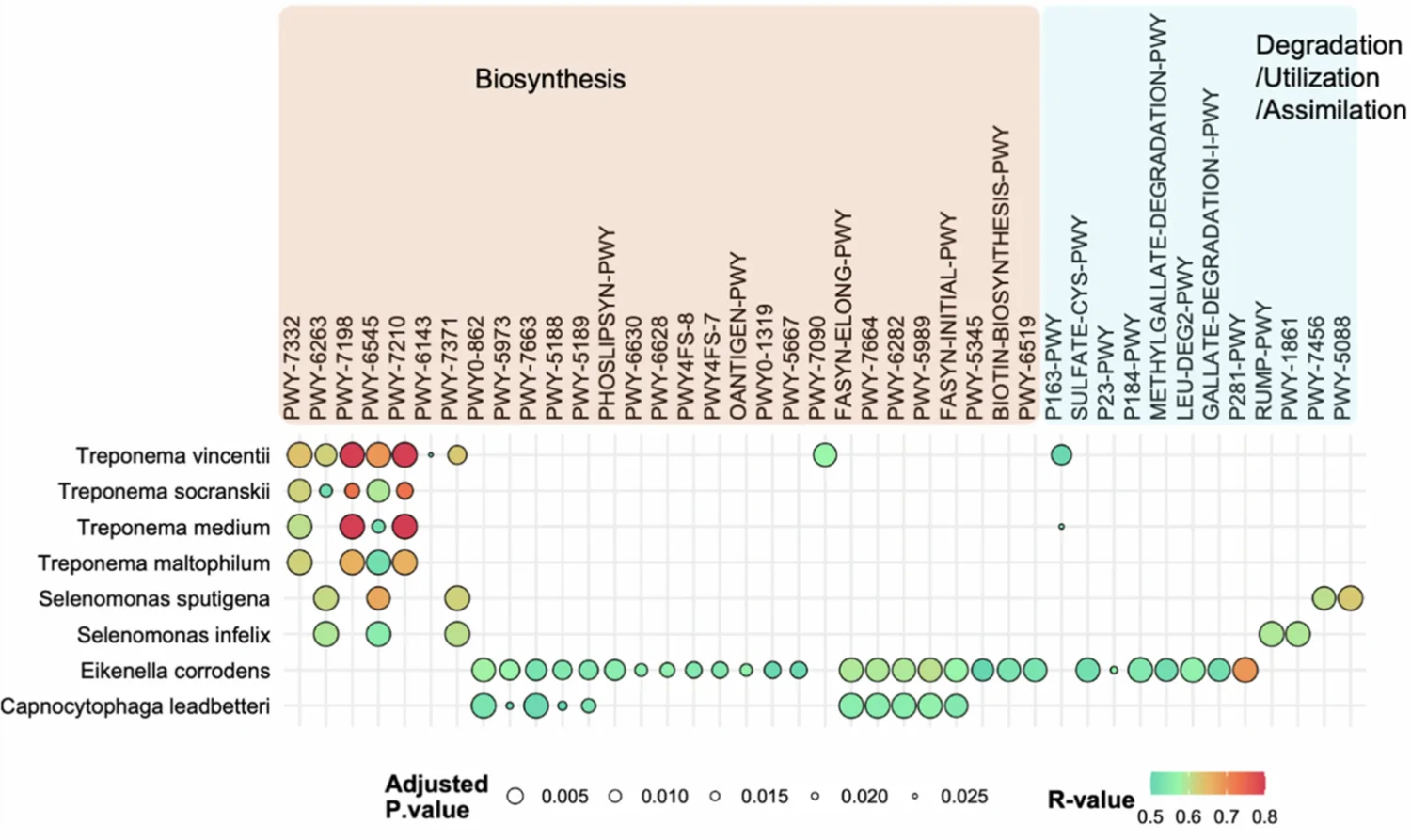
The relationships between putative species and pathways in the lesion site. The relationship with R-value > 0.5 was shown in the chart.

To bridge taxonomic shifts with potential functional consequences, we integrated our findings with predicted metagenomic profiling. We identified 143 predicted MetaCyc pathways that were significantly correlated with 25 putative species/taxa that were either differential or hub taxa (Supplementary Table 4). A notable finding was the strong positive correlation between six putative species, i.e., *Selenomonas infelix*, *Selenomonas sputigena*, *Treponema maltophilum*, *Treponema medium*, *Treponema socranskii* and *Treponema vincentii* (all enriched/hub taxa in lesions), and the PWY-6545 pathway (pyrimidine deoxyribonucleotides *de novo* biosynthesis III). In contrast, the health-associated commensal *Streptococcus oralis* was negatively correlated with this pathway. Furthermore, two other lesion-enriched putative species, *Capnocytophaga leadbetteri* and *Eikenella corrodens*, shared positive correlations with the same set of 10 distinct metabolic pathways (Figure 7).

## Discussion

The oral microbiome, the second most diverse microbial community in the human body, colonizes various niches, such as the buccal mucosa, tongue, and teeth [39]. Growing evidence suggests that dysbiosis of this community may be implicated in the pathogenesis of both benign and malignant oral diseases [40]. This study aimed to characterize the oral buccal mucosal microbiota and define its association with OSCC within a Pakistani cohort. Our analysis revealed distinct microbial profiles that clearly differentiate tumor sites, contralateral normal mucosa, and the mucosa of healthy subjects.

Our findings, while novel for a Pakistani population, align with and extend the current global understanding of the OSCC microbiome. At the phylum level, we observed a dominance of Bacillota, Bacteroidota, Pseudomonadota, Fusobacteriota and Actinomycetota, which is consistent with reports from other regions that identify these bacteria as core phyla in both healthy and diseased oral states [37,41–43]. This underscores the presence of a conserved microbial core structure within the oral cavity across diverse cohorts.

However, alterations in specific taxa demonstrated a pattern characteristic of dysbiosis. The detection of *Streptococcus oralis* as the most abundant species in healthy controls and its decline in contralateral non-lesion sites substantiates its established role as a key health-associated commensal, consistently reported to be depleted in OSCC lesions [37,44]. In contrast, the significant enrichment of *Prevotella intermedia* at tumor lesion sites in our cohort reinforces pre-existing associations between this bacterium and the development of OSCC, emphasizing its potential role as a microbial driver of tumorigenesis [42,45,46].

In addition, higher alpha diversity, partcularly in the Shannon and Shannon evenness indices, were observed at lesion sites compared with non-lesion and healthy tissues. There is emerging evidence that the oral microbiome associated with OSCC often exhibits increased, rather than decreased, microbial diversity and evenness [41,43,47]. This challenges the conventional paradigm that dysbiosis is equivalent to loss of diversity and reveals the complex, non-linear nature of the microbial shift in carcinogenesis.

All our observations, however, are not found to be uniformly supported in the literature. For example, although few researchers have reported an enrichment of Bacillota (Firmicutes) in OSCC samples (saliva and oral rinses), other studies have described its reduction in the tumor tissues compared to the healthy controls [42,48]. This variability is emphasized by our finding that Bacillota accounted for a significant proportion (33.6%) of the lesion microbiota. The existence of these differences may be due to methodological differences in sample type (saliva vs tissue vs swab) or, more importantly, to marked geographic and ethnic variations in oral microbial ecology [44,49].

The evenness in lesion sites is significantly higher, which indicates the presence of a distinct structured community, potentially indicative of dynamic interactions between microbes and the destabilization of a stable commensal-dominated hierarchy that is typical of health. Even though this expanded heterogeneity is opposite to the classical view of dysbiosis as a state of reduced stability, it is increasingly corroborated by evidence suggesting that oral microbial complexity may rise with disease progression, a finding that strongly supports our data [45,50,51]. These findings underscore novel insights into the oral microbiota of Pakistani patients with OSCC. Our results reveal a dysbiotic taxonomic landscape that, while sharing broad patterns with global studies, also exhibits distinct variations likely reflective of this specific population's unique lifestyle, environmental exposures and genetic background.

The taxonomic alteration detected in our cohort shows strong concordance with and further substantiates the established global signature of OSCC-associated dysbiosis. The significant rise in Bacteroidota (37.1% in lesions vs. 8.3% in controls), accompanied by a marked reduction in Bacillota (17.3 vs. 54.7%), substantiates international studies documenting a marked overrepresentation of Bacteroidetes and a depletion of Firmicutes in OSCC patients [49]. This sustained phylum-level alteration seems to be a hallmark of oral carcinogenesis, potentially associated with an inflammatory tumor microenvironment that may facilitate the proliferation of gram-negative taxa.

This dysbiotic trend was further explained at finer taxonomic scales. The remarkable enrichment of genera such as *Prevotella*, *Selenomonas* and *Eikenella* within lesions is consistent with previous findings linking these taxa to tumor-associated dysbiosis and inflammation-inducing activity [37,43,52–54]. In contrast, the reduction of health-associated commensals such as *Gemella* and *Streptococcus* reinforces evidence from earlier reports that species such as *Streptococcus mitis* and *S. oralis* may induce positive effects, potentially by the suppression of OSCC cell proliferation [38,55].

At the putative species level, our findings corroborate the putative tumorigenic roles of several pathogens. The enrichment of *S. sputigena, Selenomonas infelix, T. maltophilum, Treponema vincentii* and *Eikenella corrodens* conforms to their recognized associations with periodontal disease and OSCC development [49,54,56,57]. Similarly, the enrichment of *Capnocytophaga leadbetteri* underscores its potential role as an opportunistic pathogen in carcinogenesis [43].

Conversely, the significant reduction of health-associated commensals such as *S. oralis* and *S. mitis* in OSCC lesion sites highlights their potential role in maintaining oral homeostasis [38,55,58]. In healthy controls, the overrepresentation of species such as *Hoylesella pleuritidis, Haemophilus sputorum* and *Prevotella aurantiaca* further indicates their role in maintaining ecological stability [59–62]. Our results suggest that *M. salivarium* and *S. oralis* have exhibited considerable potential as microbial biomarkers for non-invasively distinguishing oral OSCC patients from healthy individuals.

Notably, the reduction of *Gemella haemolysans* in lesions within our cohort diverges from some studies reporting its elevation in OSCC [63,64]. This discrepancy may reflect important methodological, population-specific, or disease-stage differences, emphasizing that microbiota patterns in OSCC are not universal but are influenced by geographic, genetic and dietary factors [53].

The strength of our study lies in its design, which incorporates paired lesion and non-lesion sites alongside healthy controls. This approach allows for a more robust distinction of cancer-specific dysbiosis from general, health-related microbial variations. Overall, our results provide strong support for current models of bacteria-mediated carcinogenesis, wherein the enrichment of gram-negative, pro-inflammatory taxa (e.g. Bacteroidota) contributes to sustained oncogenic signaling, while the concurrent loss of protective commensals (e.g. *Streptococcus*) diminishes critical antimicrobial and immunomodulatory defenses [18,49,55]. It is important to distinguish the relative strength of evidence across our analyses. The paired lesion-versus-non-lesion comparisons, which control for inter-individual variation through the paired design and subject-stratified PERMANOVA, provide the most robust evidence for cancer-specific microbial alterations. The patient-versus-control comparisons, while informative, are more susceptible to confounding by demographic and lifestyle differences. The machine-learning findings, though internally validated by nested cross-validation, remain exploratory owing to the small healthy-control sample size (*n* = 14), class imbalance, and lack of an independent validation cohort, and should be interpreted with correspondingly graded caution.

This study provides novel insights into the microbial ecosystem of OSCC in a Pakistani cohort, delineating distinct microbial correlation patterns and functional pathway associations specific to anatomical site and disease status. Our findings both corroborate and extend the current understanding of the complex ecological networks underlying OSCC-associated dysbiosis.

A key revelation was the identification of unique hub species/taxa that are highly connected keystone taxa within the microbial networks of each site. Healthy control (HC) sites exhibited the greatest microbial complexity, with 208 species forming 1,199 correlations, a richness that was markedly reduced in both non-lesion and lesion sites. This observation aligns with the concept that a loss of microbial network stability and complexity is a feature of the dysbiotic state accompanying OSCC progression [18,48,65–67].

Notably, the positive association between *Streptococcus oralis* and *Gemella haemolysans* was conserved across all three sites. While many studies have reported the depletion of *Streptococcus* in OSCC, its persistent correlation with *G. haemolysans* may reflect their fundamental role as foundational colonizers and stabilizers of oral biofilms. This discrepancy could be attributable to population-specific factors, disease stage, or methodological differences, highlighting the need for context-specific interpretations of microbial ecology [38,50,66,68,69].

The hub species identified in healthy controls include *Corynebacterium durum*, *Streptococcus sanguinis*, *Peptidiphaga gingivicola* and *Rothia aeria*, which are consistent with taxa known to promote oral homeostasis. For instance, the synergistic interaction between *C. durum* and *S. sanguinis*, potentially mediated through fatty-acid-loaded vesicles that modulate plaque architecture, exemplifies a protective ecological mechanism that may deter pathogenic colonization [70,71].

Conversely, the emergence of *Treponema medium, T. socranskii, and T. vincentii* as hub species within lesion sites is a significant finding. This strongly aligns with growing evidence implicating spirochetes, beyond the well-studied *T. denticola*, in oral carcinogenesis [37,67,72,73]. The significant enrichment of *T. vincentii* in lesions suggests a broader pathogenic role for this genus. Mechanistically, *Treponema* species produce chymotrypsin-like proteases that can enhance epithelial invasion, disrupt host cellular barriers, and foster a pro-carcinogenic microenvironment, positioning them as potential key drivers of OSCC progression [67,72,73].

The microbial profile of non-lesion sites offered crucial insights into the potential early stages of dysbiosis. Here, *Prevotella melaninogenica* and *Segatella oulorum* were identified as hub species. While often a commensal, the keystone role of *P. melaninogenica* in non-lesion tissue may signify a very early shift towards a dysbiotic state that could predispose the tissue to carcinogenesis [59,62,68]. Similarly, the prominence of *S. oulorum*, a species linked to gingivitis and periodontal disease, reinforces the concept that clinically normal tissue in OSCC patients already resides in an intermediate dysbiotic state. This finding supports the “field cancerization” hypothesis, wherein a broader area of the oral epithelium is primed for malignant transformation [69].

Moving beyond taxonomy, predicted functional pathway analysis suggested a potential association between dysbiotic microbiota and cancer biology. Notably, there was a strong positive correlation between six lesion-enriched species, including *Selenomonas infelix, S. sputigena, Treponema maltophilum, T. medium, T. socranskii, and T. vincentii,* and the PWY-6545 pathway for *de novo* pyrimidine deoxyribonucleotide biosynthesis [15,69,74,75]. This predicted association suggests that, if functionally active, these taxa could potentially enhance local nucleotide availability to support DNA synthesis; however, this hypothesis requires validation by metagenomics, transcriptomics, or metabolomics. In stark contrast, the health-associated commensal *Streptococcus oralis* was negatively correlated with this pathway, underscoring its potential role in maintaining metabolic homeostasis and exerting anti-carcinogenic effects [38,76].

Furthermore, the co-occurrence of other pathobionts was evident. The lesion-associated species *Capnocytophaga leadbetteri* and *Eikenella corrodens* shared positive associations with the same 10 metabolic pathways, suggesting a potential association with the metabolic landscape of the tumor microenvironment. This is particularly noteworthy given the previous associations of *E. corrodens* with HPV-negative head and neck cancers [37,72]. These are PICRUSt2-predicted associations representing genomic potential, not confirmed metabolic activity.

Although our findings align with the overarching narrative of OSCC-associated dysbiosis, salient population-specific differences became evident. The persistent correlations of *S. oralis* across all sites, contrary to reports of its universal depletion, highlight the influence of cohort-specific factors such as genetics, diet, or lifestyle [66,69]. Likewise, the prominence of non-*denticola Treponema* species expands the known repertoire of spirochetes implicated in oral carcinogenesis [37,73].

These results shed light on potential diagnostic and therapeutic prospects. The identification of site-specific hub species and their associated predicted functional pathways suggests that microbial signatures could serve as candidate biomarkers for early detection or monitoring of field cancerization. Therapeutically, if validated, targeted modulation of specific bacterial metabolic pathways could represent a potential therapeutic avenue worthy of preclinical investigation [18,48,74,77].

In conclusion, there are various limitations that have to be taken into consideration. The cross-sectional nature of this study precludes the establishment of causal relationships between the microbiome and OSCC. Moreover, by focusing solely on the bacterial microbiome, we acknowledge the potential influence of viruses, fungi, and host immunity in the framing of the tumorigenic microenvironment [18]. Multi-omics-based longitudinal studies with functional validation in model systems are needed in the future in order to validate these associations and explore their translational potential. Although blank swab controls and PCR negative controls were included during DNA extraction and amplification, respectively, and yielded no detectable DNA or amplification products, these controls were not **subjected to sequencing**. Consequently, statistical decontamination of sequenced samples could not be performed, and ecological interpretations of rare, low-abundance taxa should therefore be made with caution. Additionally, species-level assignments from V3‒V4 amplicons have inherent limitations for closely related taxa (e.g., within the *Streptococcus mitis*/*oralis* group), and our findings should be validated with full-length 16S rRNA or whole-genome sequencing approaches. Furthermore, PICRUSt2-inferred pathways are hypotheses of genomic potential, not direct functional measurements, which must be validated by metagenomics, transcriptomics or metabolomics before drawing mechanistic or therapeutic conclusions. Finally, the small healthy-control sample size (*n* = 14) and lack of an independent validation cohort limit the generalizability of the machine-learning findings, which should be considered exploratory candidate biomarker discovery.

## Conclusion

This study revealed that OSCC in patients is characterized by significant oral microbiome dysbiosis, marked by the enrichment of pathogenic taxa such as *Selenomonas, Treponema, Prevotella, Eikenella* and *Capnocytophaga*, accompanied by a depletion of health-associated commensals, notably *Streptococcus* species. Although these alterations indicate broader global patterns of OSCC-associated dysbiosis, the detection of population-specific variations, such as the persistence of *Streptococcus oralis* across sites and the prominence of non-*denticola Treponema* species as hub taxa, highlights the influence of regional ecological, genetic, and lifestyle factors on microbial community structure.

Predicted functional analysis additionally suggested that species enriched in lesion sites are positively associated with metabolic pathways implicated in carcinogenesis, such as *de novo* pyrimidine biosynthesis, suggesting a potential functional association that warrants mechanistic investigation. The distinct co-abundance networks and hub species identified across lesions, contralateral non-lesion and healthy sites, reinforce the concept of field cancerization and provide novel insights into the ecological shifts preceding overt malignancy.

Collectively, our findings show that both the expansion of pro-inflammatory pathobionts and the loss of health-associated commensals emphasize the dual role of microbiome dysbiosis in OSCC development and progression. The results highlight the potential of region-specific microbial signatures as candidate non-invasive diagnostic biomarkers, warranting independent validation. To translate these findings into clinical practice, future efforts should include larger, longitudinal studies integrating multi-omics approaches to establish causality, elucidate functional dynamics, and validate host-microbe interactions in carcinogenesis. Ultimately, population-specific risk stratification, microbiome-based early detection tools, and microbiome-modulating therapies may pave the way for personalized management strategies in OSCC.

## Supplementary Material

Figure S1.tif Figure S1.tif

Figure S1.docx Figure S1.docx

Supplementary Material new Supplementary Table

Supplementary Material Supplementary data.xlsx

## Data Availability

The raw sequencing data presented are deposited in the GenBank Sequence Read Archive (SRA) under accession number PRJNA1395690.
