# Supplementary material for: Lesion-specific oral microbiome signatures and predicted carcinogenic pathways in oral squamous cell carcinoma: a paired-site study in Pakistan
Source: J Oral Microbiol. 2026 Sep 2;18(1):2721025. doi: 10.1080/20002297.2026.2721025 (PMC13539788; doi:10.1080/20002297.2026.2721025)
Supplement: Supplementary file 1 [file ZJOM_A_2721025_SM5361.docx]

**Figure S1. Rarefaction curves.** Number of observed ASVs plotted against the number of sequences (reads) for each of the 92 samples. The flattening of the curves suggests that the sequencing depth adequately captured the microbial diversity.
